# Alumni Association of Hahnemann College, Philadelphia

**Published:** 1892-04

**Authors:** W. W. Van Baun

**Affiliations:** Secretary, No. 419 Pine Street, Philadelphia, Pa.


THE ALUMNI ASSOCIATION OF THE HAHNEMANN MEDICAL COLLEGE, PHILADELPHIA, requests the pleasure of the company of the Alumni of the College at its Annual Re-union and Banquet on Tuesday, April 12th, 1892. The banquet cards, costing $3.50, can be secured from any officer of the association, The cards being limited to two hundred, the committee cannot guarantee to furnish any applied for after April 11th, 1892.

W. W. VAN BAUN, M. D., *Secretary*,
No. 419 Pine Street, Philadelphia, Pa.

